# Eosinophilic esophagitis

**DOI:** 10.1186/s13223-018-0287-0

**Published:** 2018-09-12

**Authors:** Stuart Carr, Edmond S. Chan, Wade Watson

**Affiliations:** 1 Department of Pediatrics, University of Alberta, Edmonton, AB Canada; 2 Division of Allergy & Immunology, Department of Pediatrics, University of British Columbia, Vancouver, BC Canada; 3 EoE Clinic, BC Children’s Hospital, Vancouver, BC Canada; 4 Division of Allergy, Department of Pediatrics, IWK Health Centre, Dalhousie University, Halifax, NS Canada

## Abstract

Eosinophilic esophagitis (EoE) is an atopic condition of the esophagus that has become increasingly recognized over the last 15 years. Diagnosis of the disorder is dependent on the patient’s clinical manifestations, and must be confirmed by histologic findings on esophageal mucosal biopsies. Patients with EoE should be referred to an allergist for optimal management, which may include dietary modifications and pharmacologic agents such as corticosteroids, and for the diagnosis and management of comorbid atopic conditions. Mechanical dilation of the esophagus may also be necessary. The epidemiology, pathophysiology, diagnosis, treatment, and prognosis of EoE are discussed in this review.

## Background

Eosinophilic esophagitis (EoE) is an atopic inflammatory disease of the esophagus that has become increasingly recognized in children and adults over the last 15–20 years. The disorder is sometimes referred to as “asthma of the esophagus” given that it shares many clinical and pathophysiologic characteristics with asthma [1].

Eosinophils are typically present throughout the gastrointestinal tract since it is continuously exposed to foods, environmental allergens, toxins, and pathogens. Interestingly, in healthy individuals, the esophagus is unique in that eosinophils are generally absent. In EoE, however, eosinophils infiltrate the esophagus, contributing to tissue damage and chronic inflammation. EoE is defined as a clinicopathologic disorder characterized by ≥ 15 eosinophils per high power field (HPF) in one or more esophageal biopsy specimens and the absence of pathologic gastrointestinal reflux disease (GERD) (as evidenced by a normal pH monitoring study or lack of response to adequate acid-suppression therapy) [2, 3].

The increasing number of recognized cases of EoE has resulted in a dramatic expansion of the medical literature surrounding the disease. This article provides a practical overview of recent literature surrounding the epidemiology, pathophysiology, diagnosis, treatment, and prognosis of EoE.

## Epidemiology

Given the poor awareness and recognition of the disease in the past, the epidemiology of EoE is still unclear. Current prevalence estimates in North America and Europe range from 1 to 6 per 10,000 persons [4–7]. Recent literature suggests that the prevalence of EoE is increasing [3]. The reasons for this increase are poorly understood, and although there is debate as to whether the new cases of EoE being diagnosed represent a true increase in prevalence or rather increased recognition of latent disease, increased recognition is likely not the only cause.

There are ethnic and gender variations in the prevalence of EoE, with the majority of cases reported in Caucasian males. EoE is predominant in socioeconomically developed countries, but has the highest prevalence in the United States, Western Europe, and Australia, compared with Japan and China [8]. Evidence for ethnic variation is further supported by a recent Canadian study which found a paucity of East Asian (including Chinese and Japanese) pediatric patients, compared with white and South Asian patients, in the EoE cohort [9].

## Risk factors

In addition to gender (male predominance) and race (mainly a disease of Caucasian individuals), established risk factors for EoE include atopy and other allergic conditions (e.g., allergic rhinitis, elevated serum immunoglobulin E [IgE] to common aeroallergens, asthma, and atopic dermatitis). In fact, patients with concomitant EoE and seasonal allergic rhinitis may have more EoE exacerbations during peak pollen seasons [10].

Other recognized genetic and environmental risk factors for EoE include: alterations in gut barrier function (e.g., from GERD); variation in the nature and timing of oral antigen exposure (e.g., secondary to infant feeding practices, proton pump inhibitor (PPI) use and commercial food processing); variation in the nature and timing of aeroallergen exposure (seasonal, geographic and secondary to migration); lack of early exposure to microbes and an altered microbiome (e.g., from caesarean section or lack of breast feeding) and factors relating to fibrous remodeling (e.g., *ACE* gene polymorphisms, transforming growth factor-beta [TGF-β] polymorphisms) [11, 12].

## Pathophysiology

Although the pathogenesis of EoE remains unclear, it likely results from an interplay of genetic, immune system and environmental factors as well as mechanisms of mucosal damage and fibrosis [13]. Evidence suggests that the disease is associated with T helper cell-2 (Th2) type immune responses, which are typical of other atopic conditions. In particular, elevated levels of the Th2 cytokines interleukin (IL)-4, IL-5, and IL-13, as well as mast cells, have been found in the esophageal biopsies of EoE patients [11–13]. These cytokines play an important role in the activation and recruitment of eosinophils to the esophagus. Eosinophils, in turn, play an integral role in the remodeling of esophageal tissues, which is observed histologically as subepithelial fibrosis. Eosinophils contribute to fibrosis through degranulation and secretion of their granule cationic proteins, particularly major basic protein (MBP), and elaboration of fibrogenic growth factors such as TGF-β [13].

The male predominance of EoE, as well as family history, twin concordance and genome-wide association studies, suggest that there is a genetic predisposition to EoE [11, 13]. The gene for eotaxin-3—a chemokine involved in promoting eosinophil accumulation and adhesion—has been found to be overexpressed in patients with EoE [14]. EoE has recently been found to be associated with genetic variants in calpain-14, an intracellular calcium-dependent cysteine protease that, when dysregulated, can impair esophageal epithelial barrier function [15].

EoE is also believed to represent a mixed IgE- and non-IgE-mediated allergic response to food and environmental allergens [16, 17]. IgE-mediated reactions are immediate hypersensitivity responses that usually occur within minutes after exposure to an allergen. Non-IgE mediated allergic disorders are characterized by a delayed onset (hours to days after antigen exposure), with potentially more chronic symptoms. Current thinking is that non-IgE-mediated mechanisms predominate in EoE [18]. The majority of patients with EoE have been found to have positive skin prick tests (which detect IgE-mediated reactions) and atopy patch tests (which may identify non-IgE-mediated reactions) to foods and/or aeroallergens. However, it is also clear that such testing does not accurately identify causative foods in most EoE patients [19, 20]. One small study found elevated food-specific immunoglobulin G4 (IgG_4_) in the esophageal tissue of EoE subjects compared to non-EoE controls [21]. Another study found that high-titer serum IgG_4_ to cow’s milk proteins was more common in children with EoE compared to controls [22]. Currently, the role of IgG_4_ in the pathogenesis of EoE is still unclear.

## Diagnosis and investigations

Since the physical examination of patients with EoE is often unrevealing, the diagnosis of EoE is dependent on the patient’s clinical manifestations, endoscopic assessment of the esophagus and histologic findings on esophageal mucosal biopsies.

### Clinical manifestations

Although the typical onset of EoE is in childhood, the disease can be found in all age groups, and symptoms vary depending on the age of presentation [23, 24] (see Table 1 for a summary of the clinical manifestations of EoE). Clinical manifestations in infants and toddlers generally include vomiting, food refusal, choking with meals and, less commonly, failure to thrive. Predominant symptoms in school-aged children and adolescents include dysphagia (difficulty swallowing), food impaction, and choking/gagging with meals, particularly while eating foods with coarse textures. Other symptoms in this patient population include abdominal/chest pain, vomiting, and regurgitation. A careful history in children and adolescents with EoE reveals that they have learned to compensate for these symptoms by eating slowly, chewing excessively or taking small bites, drinking excessively with meals, lubricating meals inordinately with sauces, and avoiding specific food consistencies such as meats (or other foods with coarse textures) [25, 26].

**Table 1:** Clinical manifestations of EoE

	Infants/toddlers	Children	Adults
**Symptoms**	• Feeding aversion/intolerance • Vomiting • Food refusal • Choking with meals • Failure to thrive • Sleep disturbance	• Dysphagia • Choking/gagging with coarse textures • Food impactions • Abdominal/chest pain • Throat pain • Vomiting/regurgitation • Nausea • Sleep disturbance • Decreased appetite	• Dysphagia (predominant) • Food impactions • Food avoidance • Intractable heartburn • Regurgitation • Retrosternal pain • Chest pain
**Response to acid-suppression therapy**	Poor	Poor	Poor
**Associated conditions**	• Food allergy • Atopic dermatitis	• Asthma • Allergic rhinitis • Food allergy	• History of atopy • Asthma • Allergic rhinitis

The predominant symptom in adults is dysphagia; however, intractable heartburn and food avoidance may also be present. Due to the long-standing inflammation and possible resultant scarring that has gone unrecognized, adults presenting with EoE tend to have more episodes of esophageal food impaction as well as other esophageal abnormalities such as Schatzki ring (a narrow ring of tissue located just above the junction of the esophagus and stomach), esophageal webs (small, thin growths of tissue that partially block the esophagus) and, in some cases, achalasia (an esophageal motility disorder characterized by difficulty swallowing and regurgitation). However, it is important to note that some patients with EoE are asymptomatic, and suspicion of the disease is based upon incidental findings at endoscopy that is performed for other indications or upon evidence of food impaction.

Although many of these symptoms overlap with GERD, the majority of patients with EoE exhibit a poor response to acid-suppression therapy (e.g., PPIs), and up to 75% have a personal or family history of atopic disease (e.g., asthma, eczema, allergic rhinitis and/or food allergies) [23]. However, it is also important to note that up to one-third of patients with endoscopic and symptomatic features of EoE will respond to PPI monotherapy, and this disease entity is referred to as PPI-responsive esophageal eosinophilia (PPI-REE) [3]. Whether PPI-REE represents an independent clinical disorder or whether it is a subtype of EoE or GERD remains an area of controversy [27–33]. However, recent evidence suggests that it shares important similarities with EoE, including the pathogenic allergic mechanisms that underlie the disease [3]; further research on the pathophysiology of PPI-REE is needed.

### Endoscopy

**Fig. 1:**
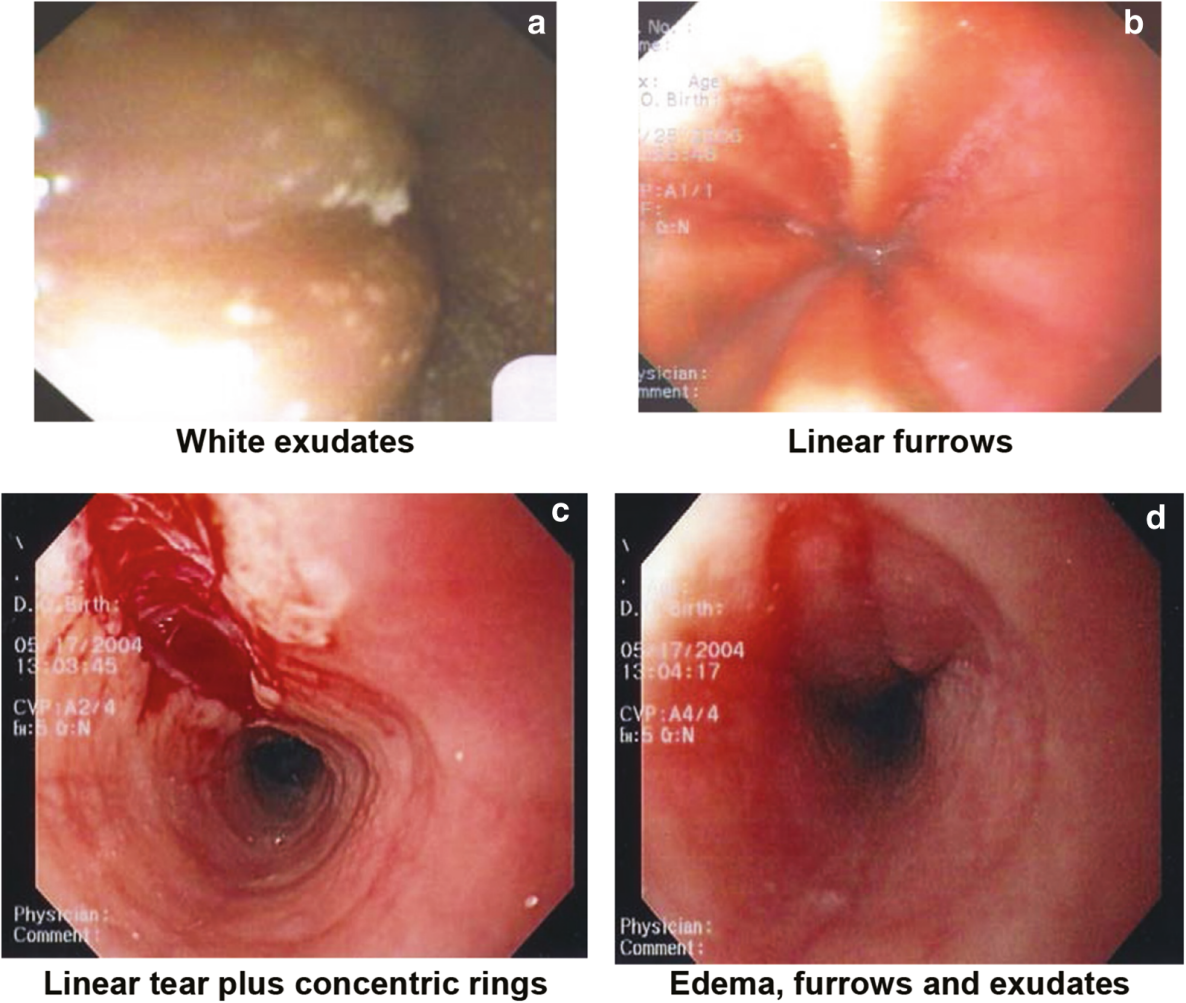
**Images of endoscopic features of EoE**.** a** **White exudates** Courtesy of Dr. Hien Huynh.** b** **Linear furrows** Courtesy of Dr. Hien Huyn.** c** **Linear tear plus concentric rings** Courtesy of Dr. Adrian Jones.** d** **Edema, furrows and exudates** Courtesy of Dr. Adrian Jones

Although the endoscopic examination may be unremarkable, endoscopic features of EoE have been well-characterized and include: linear furrowing (ridges or furrows in the esophageal wall), concentric rings, white speckled exudates (eosinophilic abscesses), Schatzki ring, small-calibre esophagus, and linear superficial mucosal tears that occur after introduction of the endoscope [3]. Table 2 provides a more detailed description of each of these features. Images of exudates, linear furrows and tears are provided in Fig. 1.

**Table 2:** Endoscopic features of EoE

Endoscopic feature	Description
Linear furrowing	• Vertical esophageal lines or ridges in the esophageal wall
Concentric rings	• Multiple rings that may be fine, web-like or thickened (also termed the “corrugated” or “ringed” esophagus)
White speckled exudates	• Patches of whitish papules (1–2 mm in diameter) • Resembles esophageal candidiasis
Schatzki ring	• Narrow ring of tissue located just above the junction of the esophagus and stomach
Small-calibre esophagus	• Narrowed esophagus, with fixed internal diameter • Featureless, unchanging column • Poor expansion on air insufflation • Proximal and/or distal stenosis
Linear superficial mucosal tears	• Mucosal abrasions or shearing that occur upon minimal contact (e.g., after simple passage of a routine endoscope)

Patients found to have signs of EoE on endoscopy (performed acutely for food impaction or for other reasons) should undergo an empiric 8-week trial of high-dose PPI therapy (twice daily) before repeat endoscopy in order to rule out GERD or PPI-REE. A barium swallow may also be considered in severely symptomatic patients prior to endoscopy to rule out severe small-calibre esophagus.

Although endoscopic findings are helpful in identifying patients with EoE [34], they are not diagnostic of the disease in the absence of pathognomonic clinical symptoms. Additionally, it is important to rule out esophageal candidiasis when white exudates are identified. As such, all patients with suspected EoE must undergo esophageal mucosal biopsies to confirm the diagnosis.

### Esophageal mucosal biopsies

Currently, endoscopic mucosal biopsy remains the most important diagnostic test for EoE, and is required to confirm the diagnosis. Biopsy specimens from both the proximal or mid and distal esophagus should be obtained regardless of the gross appearance of the mucosa, as well as from areas revealing endoscopic abnormalities [2]. At least four biopsies are required to obtain adequate sensitivity for the detection of EoE (5–6 biopsies are generally recommended). One study found that six biopsies increase the sensitivity of this investigation to 99% [35].

As discussed earlier, a definitive diagnosis of EoE is based on the presence of at least 15 eosinophils/HPF in the esophageal biopsies of patients despite treatment with high-dose PPI. GERD can increase eosinophilic infiltration in the distal esophagus, however, eosinophils associated with GERD generally occur at a lower density (i.e., < 15/HPF). Also, those patients suspected of having EoE based on the initial biopsies who respond clinically and histologically to PPI monotherapy should be diagnosed as having PPI-REE.

### Allergy assessment

A thorough personal and family history of other atopic conditions is recommended in all patients with EoE. Testing for allergic sensitization may be considered, with skin prick testing or blood testing for allergen-specific IgE. This is particularly important for the 10–20% of EoE patients who also have symptoms of immediate IgE-mediated food allergy [20]. Current methods of food allergy testing, which identify IgE-mediated sensitization, may not identify EoE triggers [20, 36, 37]. Therefore, physicians should ideally discourage allergy testing if the patient is eating foods without a history of immediate reactions. Instead, testing may be considered for expanding an already restricted diet, being very careful to avoid over-testing and over-interpretation.

Atopy patch testing has been used in some centres for the potential identification of delayed, non-IgE (cell-mediated) reactions. It is similar to patch testing for contact dermatitis and involves placing a small quantity of an allergen directly on the skin and then examining for a local, delayed reaction after a specified time (48–96 h). Although some studies suggest that atopy patch testing combined with skin prick testing may be able to more accurately identify EoE trigger foods in children [17], this test has not yet been standardized, and the positive predictive value remains poor [38]. In addition, such testing has not been shown to be as helpful in adult EoE patients. More studies are needed to assess the reliability and validity of atopy patch testing before it can be recommended for routine use in the diagnosis and monitoring of EoE.

## Treatment

**Fig. 2:**
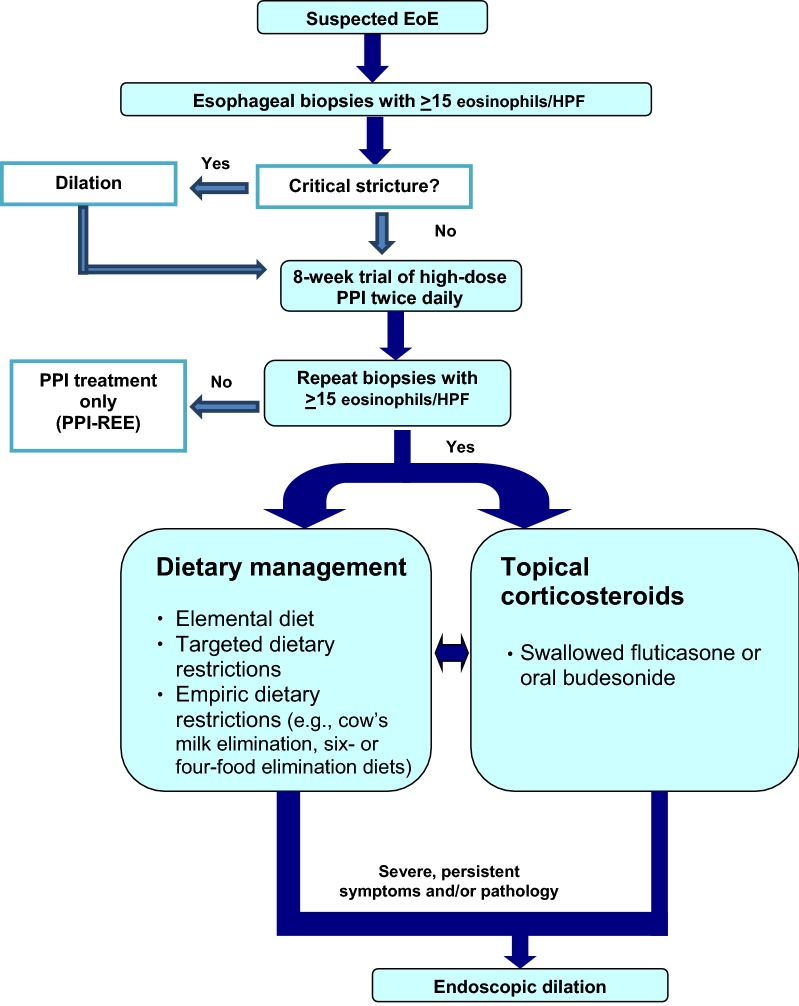
**Simplified algorithm for the diagnosis and management of EoE [**39**]**. *EoE* eosinophilic esophagitis, *PPI* proton pump inhibitor, *GERD* gastrointestinal reflux disease, *HPF* high power field, *PPI-REE* proton pump inhibitor responsive esophageal eosinophilia

Treatment strategies available for EoE fall into three categories: (1) avoidance of triggers through dietary modification, (2) pharmacologic therapy (predominantly corticosteroids), and (3) mechanical dilation of the esophagus. A simplified algorithm for the diagnosis and management of EoE is shown in Fig. 2 [39].

### Dietary management

Three dietary approaches for the management of EoE have emerged: (1) the elemental diet, (2) empiric dietary restrictions (e.g., cow’s milk elimination, or the six- or four-food elimination diets), and (3) targeted dietary restrictions based on allergy testing. The elemental diet involves the removal of all sources of potentially allergenic protein from the patient’s diet through the use of an amino acid-based formula for nutritional support. Assuming there is a favorable clinical and histologic response, one new food per week is reintroduced in a sequential fashion, beginning with the least allergenic foods (fruits and vegetables) to the most highly allergenic (e.g., dairy, soy, egg, wheat, and peanuts). A repeat endoscopic assessment is performed after the reintroduction of every 3–5 foods to ensure that the inflammation has not recurred.

Although the elemental diet is associated with high rates of clinical and histologic improvement in children with EoE (i.e., > 90%), symptoms often recur after normalization of the patient’s diet [17, 40]. Furthermore, given the unpalatable taste of the formula, most patients require feeding by nasogastric tube which may lead to adherence issues and impaired quality of life (QoL), particularly in adolescents and adults. In this latter population, the elemental diet is only effective in approximately 70% of patients [41].

Targeted and empiric dietary restrictions are often employed before considering an elemental diet. These restrictions involve the elimination of foods based on results of skin prick and atopy patch testing. Current studies suggest a success rate of 50–70% for targeted, test-driven diets in children, and even lower success rates in adults [42]. Furthermore, clinically-irrelevant positive results and false negative results complicate this dietary approach. Therefore, further studies examining both the positive and negative predictive value of targeted dietary restrictions in EoE are necessary.

Rather than basing dietary elimination on skin prick testing and atopy patch testing, empiric dietary restrictions involve the elimination of the most common allergenic foods (in the absence of, or regardless of, the results of allergy testing). Some centres currently choose cow’s milk elimination as the first trial, based on data suggesting a response rate approaching that of the six-food elimination diet (~ 65%) but with greater convenience/feasibility [43, 44]. For patients in whom cow’s milk elimination is insufficient, there are two other approaches: the six-food elimination diet (dairy, eggs, wheat, soy, peanuts/tree nuts, and fish/shellfish) [45], or the four-food elimination diet (dairy, eggs, wheat, and legumes, as studies suggest tree nuts, fish, and shellfish are less commonly implicated in EoE) [46]. Dairy is clearly the most commonly implicated triggering food (74%), followed by wheat (26%) and egg (17%). In one large pediatric cohort, eliminating meats (beef and chicken) in addition to dairy, eggs, wheat, and soy increased the response rate to 77% [37]. A systematic review of empiric elimination diets suggests an overall success rate of this approach of approximately 70% [47].

With all dietary approaches, it remains unclear how long specific foods need to be avoided, which order to reintroduce individual foods, and how often to perform gastroscopy and mucosal biopsies for reassessment. Recent reports also suggest that there may be an increased future risk of anaphylaxis in those who restrict foods for a prolonged period, suggesting loss of tolerance [48–50]. Clearly, more studies on this approach are necessary, including an attempt to evaluate patient QoL given the extensive dietary restrictions often required that involve many “staple” foods. Furthermore, if several foods are to be eliminated simultaneously, enlisting the assistance of a dietitian may be beneficial, particularly in the pediatric population. This may help ensure nutritional requirements are met in order to facilitate adequate growth and development.

### Pharmacologic management

Medical therapy for EoE primarily focuses on corticosteroids. Systemic (oral) corticosteroids were one of the first treatment options shown to be effective in patients with EoE. Both clinical and histologic improvement have been noted in approximately 95% of EoE patients using systemic corticosteroids; however, upon discontinuation of therapy, 90% of patients experience a recurrence in symptoms [51]. Furthermore, given that prolonged use of systemic corticosteroids is associated with well-known and potentially serious adverse effects, their long-term use is not recommended. Systemic corticosteroids should be reserved for emergent cases such as patients with dysphagia requiring hospitalization or patients experiencing significant weight loss or dehydration due to swallowing difficulties.

Given their substantially better safety profile, topical corticosteroids delivered to the esophagus have become the mainstay of pharmacotherapy for patients with EoE. Both swallowed fluticasone propionate (500–1000 µg/day) and oral viscous budesonide (1000–2000 µg/day) have been shown to be effective in the management of EoE [2, 3]. Fluticasone propionate is delivered via a pressurized metered dose inhaler (pMDI) that is activated into the mouth (without inhaling and without a spacer device) and swallowed. Budesonide is administered orally after the contents of a vial used for nebulization are mixed with a thickening agent to increase the viscosity of the solution which, theoretically, slows its transit over the esophageal lining [23]. A variety of sweeteners/vehicles can be chosen for increasing viscosity, including sucralose, applesauce, and honey [52].

Randomized clinical trials of topical fluticasone propionate therapy have shown both histologic and symptomatic improvements in 50–80% of pediatric and adult patients with EoE [53, 54]. The most frequent complications noted with topical fluticasone propionate are oropharyngeal and esophageal candidiasis. Oral viscous budesonide has emerged as a more convenient and successful treatment option, with high clinical and histologic response rates in pediatric and adult patients with EoE [55–61]. Oral budesonide has also been associated with a lower risk of developing esophageal candidiasis.

A recent meta-analysis confirmed the effectiveness of topical corticosteroids in the treatment of EoE, with minimal adverse effects and no evidence of adrenal suppression [62]. However, other studies have described the potential for adrenal suppression with either fluticasone or budesonide, ranging from approximately 10–65% of patients treated with these agents [63–65]. However, how to effectively screen for adrenal suppression, given the lack of specific symptoms and limited access to low-dose adrenocorticotropic hormone (ACTH) stimulation testing, continues to be a topic of debate.

Patients using topical corticosteroids for EoE should be advised not to eat, drink, or rinse their mouth for 30 min after using the medication. After 6–8 weeks of topical therapy, patients should undergo repeat endoscopic assessment to ensure histologic response to therapy. If a therapeutic response is confirmed, treatment should be reduced to the lowest effective dose with appropriate follow up. It is important to note that symptoms and pathological changes often recur after discontinuation of topical corticosteroids. Therefore, many patients with EoE will require long-term treatment.

There are few other medical treatment options for EoE. Although a small study of eight patients with EoE found a significant improvement in symptoms in the majority of subjects receiving the leukotriene receptor antagonist (LTRA), montelukast, no improvement in histology was noted [66]. A subsequent prospective trial of montelukast failed to reproduce this symptomatic improvement, and again failed to elicit any histologic response [67]. Accordingly, LTRAs are not recommended for the treatment of EoE. Although one small study examining the use of immunosuppressive agents (azathioprine and 6-mercaptopurine) for the treatment of adult EoE patients showed promising results [68], this potential therapeutic option has not yet been evaluated further.

Given that IL-5, IL-13 and IgE appear to play a role in the pathogenesis of EoE, humanized monoclonal antibodies against IL-5 (reslizumab, mepolizumab, benralizumab), IL-13 (RCP4046) and IgE (omalizumab) have been proposed as potential therapeutic options for the disease. Although anti-IL-5 antibodies have been shown to decrease esophageal eosinophil counts in patients with EoE, to date these agents have failed to lead to histologic remission or significant clinical improvements [69–71].

The anti-IgE antibody, omalizumab, is used for the management of severe atopic asthma and allergic rhinitis. Since omalizumab has been shown to lower eosinophil counts in the blood and lungs of patients with asthma [72], it has also been proposed as a potential therapeutic approach for EoE. To date, however, results with omalizumab in patients with EoE have been mixed [73, 74]. As such, this biologic agent is not currently recommended for management of the disease.

Among the biologic agents studied in EoE, RCP4046 (an anti-IL-13 monoclonal antibody) and dupilumab (a fully human monoclonal antibody directed against the alpha subunit of the IL-4 receptor) appear to hold the most promise as novel therapeutic options for the disease [75–77]. A small, randomized controlled trial of adult patients with EoE found significant improvements in mean esophageal eosinophil count and improved endoscopic features with RCP4046 [77]. A phase 2 study comparing dupilumab to placebo in adults with active moderate-to-severe EoE showed significant improvements in dysphagia with weekly dupilumab treatment [76]. Esophageal eosinophil counts, esophageal distensibility, and endoscopic and histopathologic measures of disease severity were also improved with dupilumab treatment.

### Endoscopic dilation

Esophageal endoscopic dilation is most commonly used in adults with established esophageal strictures. Although many physicians are fearful to dilate EoE patients due to concerns regarding mucosal tears and perforations, numerous case series attest to the safety and efficacy of esophageal dilation [78], with many patients experiencing symptom relief for an average of 2 years. Furthermore, mucosal tears are actually a sign of successful dilation, not complications. According to recent EoE consensus statements and societal guidelines, periodic dilation is now considered an acceptable alternative to medical or dietary therapy in some healthy adults with EoE [3, 78].

## Prognosis

The long-term prognosis for patients with EoE is unknown. Some patients may follow a “waxing and waning” course characterized by symptomatic episodes followed by periods of remission. There have also been reports of apparent spontaneous disease remission in some patients; however, the risk of recurrence in these patients is unknown. It is possible that long-standing, untreated disease may result in esophageal remodeling, leading to strictures, Schatzki ring and, eventually, achalasia. According to a recent review on the natural history of EoE, progressive remodelling appears to be gradual, but not universal. Also, the duration of untreated disease appears to be the best predictor of stricture risk [79].

To date, neither dietary elimination nor medical therapy has been shown to modify the natural history of EoE [80]. Therefore, maintenance therapy and/or periodic esophageal dilation are important considerations given that the majority of patients with this disease will develop recurrent symptoms and esophageal eosinophilia upon cessation of medical or dietary therapy.

Furthermore, although the natural history suggests that EoE is a chronic, recurrent disease [81], it appears benign and is not associated with a risk of malignancy [80]. More studies are needed to better understand the natural history of EoE.

## Conclusions

EoE is an evolving condition that requires further study to better understand the mechanisms of disease development and tissue injury, natural history, and optimal management. Although clearly an atopic condition, our ability to identify specific allergic triggers remains limited, and this is an important focus of ongoing investigation. As our understanding surrounding EoE improves, so will strategies for the diagnosis and treatment of the condition.

## Key take-home messages

- EoE is an atopic condition of the esophagus that has become increasingly recognized over the last two decades.
- Endoscopic mucosal biopsy revealing ≥ 15 eosinophils/HPF in one or more specimens remains the most important diagnostic test for EoE, and is mandatory for diagnosis.
- Patients with EoE should be referred to an allergist to help identify potential triggers, optimize treatment, and manage concurrent atopic conditions.
- Skin or specific IgE blood testing for foods has relatively low value for identifying food triggers of EoE due to inadequate positive and negative predictive value, and ideally should be reserved for confirming potentially anaphylactic IgE-mediated food allergy.
- The elemental diet, empiric dietary restrictions and targeted dietary restrictions are associated with high rates of clinical and histologic improvement in patients with EoE.
- Topical corticosteroids delivered to the esophagus are the mainstay of pharmacotherapy for patients with EoE.
- Esophageal endoscopic dilation is most commonly used in adults with established esophageal strictures; periodic dilation is now considered an acceptable alternative to medical or dietary in some healthy adults with EoE.

## Abbreviations

EoE: eosinophilic esophagitis
GERD: gastrointestinal reflux disease
HPF: high power field
IgE: immunoglobulin E
PPI: proton pump inhibitor
TGF-β: transforming growth factor-beta
PPI-REE: proton pump inhibitor-responsive esophageal eosinophilia
ACTH: adrenocorticotropic hormone
QoL: quality of life
Th2: T-helper cell 2
IgG_4_: immunoglobulin G4
pMDI: pressurized metered dose inhaler
MBP: major basic protein
